# Sugar Content and Warning Criteria Evaluation for Popular Sugar-Sweetened Beverages in Taipei, Taiwan

**DOI:** 10.3390/nu14163339

**Published:** 2022-08-15

**Authors:** Chieh Yen, Ya-Li Huang, Mei Chung, Yi-Chun Chen

**Affiliations:** 1 School of Nutrition and Health Sciences, Taipei Medical University, Taipei 110, Taiwan; 2 Nutrition Division, Far Eastern Memorial Hospital, New Taipei City 220, Taiwan; 3 Department of Public Health, School of Medicine, College of Medicine, Taipei Medical University, Taipei 110, Taiwan; 4 School of Public Health, College of Public Health, Taipei Medical University, Taipei 110, Taiwan; 5 Friedman School of Nutrition Science and Policy, Tuft University, Medford, MA 02115, USA

**Keywords:** sugar-sweetened beverage, energy, sugar content, Chilean warning label system, WHO guideline

## Abstract

Sugar intake may increase the risk of obesity, cardiovascular disease, diabetes, and dental caries. In Taiwan, people frequently consume sugar-sweetened beverages (SSBs). This study explored the energy and sugar content of Taiwanese SSBs and evaluated them using the Chilean warning label system (>70 kcal/100 mL and >5 g sugar/100 mL) and the World Health Organization (WHO) sugar guideline (≤25 g sugar). A total of 341 SSBs with volumes ≤600 mL were analyzed. No significant differences were observed in sugar per serving among different types of SSBs, but a great variation in portion size (i.e., package size for individual consumption) was noted. The energy and sugar ratios per serving were lower in soft drinks and coffee and tea containing >1 serving than in those containing only one serving. The calorie and sugar ratios per portion were higher in all types of SSBs containing >1 serving per portion than in those containing exactly one serving. Approximately 70.0% of Taiwanese SSBs were classified as high sugar according to the Chilean criteria, and 41.6% of SSBs exceeded the WHO guideline. Moreover, 40.8% of SSBs that were not considered as high sugar according to the Chilean criteria contained >25 g sugar per portion. For individual consumption, it is more clear that nutrition labeling is based on portion rather than serving. Evaluating SSBs on sugar/portion rather than sugar/100 mL will help consumers make better choices.

## 1. Introduction

Excess sugar consumption is associated with increased risk of obesity, type II diabetes, and dental caries [1,2]. According to the 2013–2016 Nutrition and Health Survey in Taiwan, 45.4% of adults aged 19 years and older were either overweight or obese, and over 30% of Taiwanese people consumed sugar-sweetened beverages (SSBs) at least once a day [3]. The World Health Organization (WHO) has reported that SSBs are the main cause of excess sugar intake and increased health risks, such as obesity, dental caries, lower nutrient density, and chronic diseases. Accordingly, the WHO recommends that daily sugar intake should not exceed 10% of the total daily energy intake and should be reduced to below 5% when possible [4].

Nutrition labeling and front-of-package (FOP) labels, including warning labels of sugars, constitute a widely promoted public health strategy. Labels are the primary means of educating consumers about food product ingredients, thereby enabling them to choose healthy options [5]. FOP labels provide consumers with concise, easy-to-understand information about the nutrient profile of a food product [6]. The WHO endorses FOP nutrition labels as a crucial policy to inform consumers, encourage them to reevaluate their diets and make healthier food choices, and reduce consumption of products with excessive sugar [7]. However, current FOP labels vary in their degree of regulation. For example, FOP nutrition labeling is voluntary in the UK, Australia, and the European Union [8]. In 2016, Chile became the first country to implement a mandatory national FOP nutrient warning label policy [9]. Chile requires “high in” warning labels for products that exceed the established limit for sodium, saturated fats, total sugars, or total energy [10]. Several other Latin American countries as well as Israel and Canada are in the process of developing or implementing overarching warning label systems [11]. On a per-product basis, SSBs have also been targeted for labeling. In California, legislative bills have been introduced that require SSBs to display health warning labels on product containers, similar to tobacco warning labels [12]. These labels carry the text, “Drinking beverages with added sugar(s) contributes to obesity, diabetes, and tooth decay” [12].

Although food and beverage reformulation has been suggested as a key strategy for obesity prevention, little evidence exists on how real-life initiatives encourage reformulation. A prospective study found that the most frequent reductions were in the proportion of “high in sugar” warning labels on beverages and milk/milk-based drinks after implementation of the Chilean Food Labeling and Advertising Law [13]. UK Soft Drinks Industry Levy motivated many manufacturers to reduce sugar content in soft drinks, which could reduce people’s exposure to liquid sugars and related health risks. Food and beverage warning label policies are increasingly popular globally [14]. Two types of warning labels have been proposed: nutrient warning labels (messages that alert consumers that a beverage has a high amount of a harmful nutrient) and health warning labels (messages that describe potential health hazards of a particular beverage) [10,12]. Many countries, including Peru, Uruguay, Mexico, and Israel, have already passed or implemented warning label policies to reduce SSB intake, and similar policies are under consideration in Brazil, Canada, and South Africa [15]. Chile’s Food Labeling and Advertising Law is considered by the WHO to be the most ambitious and restrictive law implemented in the fight against obesity [16,17]. Under this law, regulated foods and drinks are required to carry warning labels when the nutrients exceed thresholds. A review study reported that most of studies on food labeling focused on the effects of FOP labels on consumer behavior, whereas few studies explored the total energy and total sugar content of SSBs [18]. Nutrition labeling and warning labels are mainly based on per serving. Where SSBs sold in larger bottles can be consumed as one serving, the nutrition label recommends two to three servings per bottle [19]. Thus, this study compared the energy and sugar content by serving, concentration, and portion (i.e., package size for individual consumption) of SSBs.

Culture plays an important role in the types of SSBs consumed. Carbonated drinks, including sodas, are common in Western countries, whereas coffee and tea drinks are more common in Asia [20,21]. A few Western studies have revealed the sugar content of various SSBs in their local markets, such as carbonated SSBs in the UK [22], fruit drinks and carbonated drinks in New Zealand [23], and soft drinks in Australia [24], but these studies were region specific and lacked data for coffee and tea drinks. Accordingly, the present study investigated the energy and sugar content of SSBs, including coffee and tea drinks, available in Taiwanese supermarkets and convenience stores and examined the applicability of warning criteria.

## 2. Materials and Methods

### 2.1. Data Collection

Content analysis was used to investigate the energy and total sugar content of SSBs as stated on nutrition labels and to evaluate the applicability of warning labels for SSBs. We included SSBs with a volume of ≤600 mL per individual portion [25]. All SSBs were classified into 4 major categories and 10 subcategories in accordance with the product classification standards set by Taiwan’s Ministry of Economic Affairs [26,27]. We examined (1) soft drinks, comprising carbonated soda, coffee drinks, sports drinks, and traditional drinks; (2) protein drinks, comprising flavored milk, fermented milk, and soy/rice milk; (3) fruit drinks, comprising fruit juice and fruit/vegetable drinks; and (4) coffee and tea, comprising sweetened coffee, sweetened tea, and milk tea. Sparkling/mineral/flavored water products, which have no sugar content, were excluded. Researchers collected data from November 2017 to April 2019 at chain supermarkets (e.g., PXMart and Carrefour) and convenience stores (e.g., 7-Eleven and Family Mart) in Taipei City. SSBs were identified by viewing all products in the stores, and the packaging information for each SSB was photographed for subsequent analysis. This study did not involve any human participants, and all data we obtained were publicly available; therefore, ethics committee approval was not required.

### 2.2. Coding Procedures

The first author developed a coding form and then instructed two researchers in the coding procedure. Both researchers fully understood the coding procedure and contributed equally to the coding of photographed information using a uniform technique. The coded components of food packaging information included general information, product category, portion size, and the nutrition fact label, as detailed in Table 1 [28]. To group beverages, we used an SSB classification system that was previously developed for US and Chilean markets and adapted it for Taiwan [29,30].

### 2.3. Nutrition Labels of SSBs

**Figure 1:**
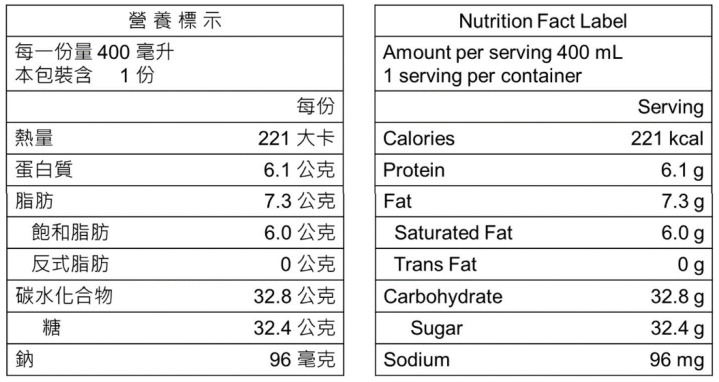
Nutrition fact labels in Traditional Chinese (**left**) and English (**right**).

For each product, the nutrition fact label for one serving was coded and then used to calculate the energy and nutritional content per portion. Portion size is defined as the total volume of beverage. Although Taiwan’s Ministry of Health and Welfare suggests 240 mL as a reference serving size [31], the serving sizes among similar foods were not necessarily the same (see Appendix A). Figure 1 presents the nutritional information commonly found on nutrition fact labels. Energy and nutrients are presented on a per-serving basis [32].

### 2.4. Energy and Sugar Criteria for SSBs

Three evaluation methods were used to assess the energy and sugar content for different categories of SSBs. The Chilean criteria for high calorie (SSBs containing >70 kcal/100 mL [10]) and high sugar (SSBs containing >5 g sugar/100 mL [10]) provided an acceptable ratio for energy and sugar content, while the WHO sugar guideline allowed us to assess sugar content by the whole package (=portion size). SSBs containing >25 g sugar per portion were defined as exceeding the WHO guideline. The WHO recommends limiting free sugar intake to 25 g/day to reduce the risk of noncommunicable diseases [4]. WHO defines the term “free sugars” as all monosaccharides and disaccharides added to foods by the manufacturer, cook, or consumer, plus sugars that are naturally present in honey, syrups, and fruit juices. Hence, we chose 25 g of sugar as a cutoff point.

### 2.5. Coding Reliability and Data Analyses

We used kappa statistical tests to measure the interrater reliability of the two coders. Kappa coefficients were calculated by analyzing the final coded data for 50% of SSB packages. A coefficient of 0.61–0.80 indicated substantial agreement between the coders, and 0.81–1.00 indicated almost perfect agreement [33]. Data with kappa coefficients >0.80 were considered acceptable. All data were analyzed using SPSS version 18.0 (SPSS, Chicago, IL, USA). Descriptive statistics were used for frequencies (*n*), percentages (%), and median values (interquartile range (IQR)). Coefficients of variation (CVs) were calculated using the standard deviation divided by the mean to obtain a standard measure of variation across SSB categories. The Kolmogorov–Smirnov test was used to examine the normal distributions of serving sizes, portion sizes, energy, and nutrient and sugar content to determine whether any of these variables had abnormal distributions. The Wilcoxon signed rank test was used to compare the serving size and portion size in each SSB category. The Kruskal–Wallis test was used to compare the energy and nutrient content of different types of SSBs. The Mann–Whitney *U* test was used to compare the energy and sugar content between products containing one serving and those containing >1 serving in each SSB category. Statistical significance was set at *p* < 0.05 for all data analyses.

## 3. Results

### Nutrition Content, Serving Size, and Portion Size

We collected 341 SSBs representing different types of soft drinks (*n* = 72), coffees and teas (*n* = 117), protein drinks (*n* = 107), and fruit drinks (*n* = 45). Table 2 reveals the nutrition fact information of the SSBs. Due to higher protein and fat content, protein drinks contained more energy per serving than the other SSBs (*p* < 0.001). No significant differences in sugar per serving and carbohydrates per serving were observed among different SSBs.

**Figure A1:**
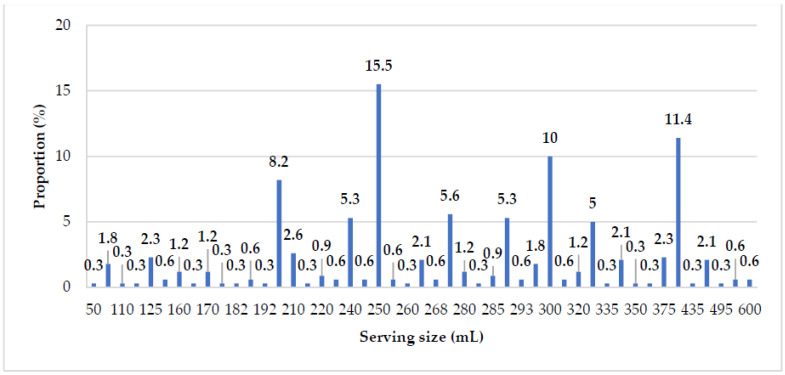
Distribution of serving sizes for sugar-sweetened beverages (*N* = 341).

Serving sizes varied drastically among the SSBs. We counted 47 different serving sizes ranging from 50–600 mL, with 200, 250, 300, and 400 mL all being common sizes and each representing more than 8% of the total distribution (see Appendix A, Figure A1). Table 3 presents the variations in serving sizes and portion sizes. Portion sizes of soft drinks and coffee and tea were significantly larger than serving sizes. Soft drinks had larger serving sizes than the other SSBs, whereas protein drinks and fruit drinks had smaller serving sizes than the other SSBs. CVs for serving size ranged from 19.0% to 36.7% across all SSB categories. Soft drinks exhibited relatively little variation (CVs of <20%) for serving size, whereas protein drinks and fruit drinks exhibited variation (CVs of >30%). Portion sizes for all SSBs exhibited variation (CVs of >30%) and portion sizes exhibited greater variation than serving sizes.

**Figure 2:**
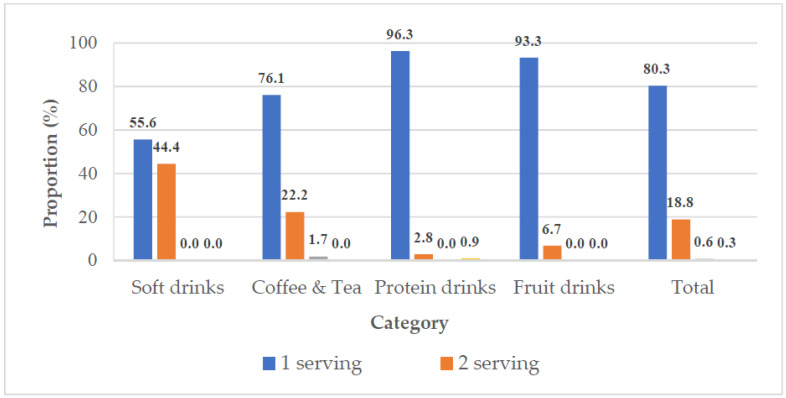
Distribution of servings per portion among different types of SSBs.

Figure 2 depicts the distribution of number of servings per portion. Approximately 20% of SSBs contained >1 serving per portion. A total of 44.4% of soft drinks and 23.9% of coffee and tea contained >1 serving per portion. Less than 10% of protein drinks and fruit drinks contained >1 serving.

Table 4 reveals the differences in energy and sugar content between SSBs containing a single serving per portion and those with >1 serving per portion. Single-serve soft drinks and coffee and tea contained more energy per serving than those with >1 serving. However, single-serve soft drinks and coffee and tea contained less energy per portion than those with >1 serving. No significant differences were found for energy per serving between single-serve protein drinks and fruit drinks and those with >1 serving. Single-serve protein drinks and fruit drinks contained less energy per portion than those with >1 serving. A similar trend was found in comparisons of sugar content among the different types of SSBs. All SSBs with >1 serving had significantly higher sugar per portion than those containing a single serving.

**Figure 3:**
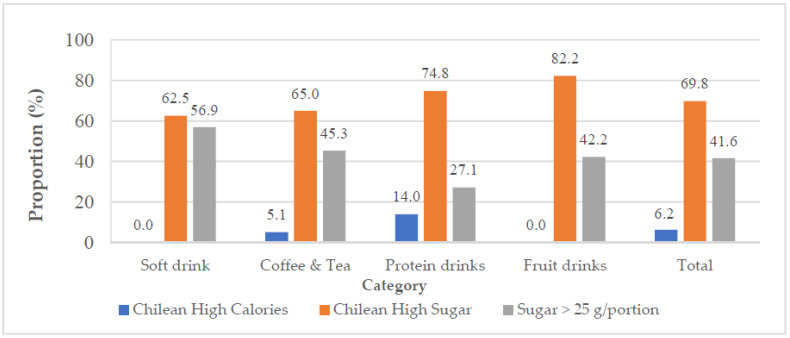
Distribution of SSBs classified as high calorie or high sugar according to Chilean criteria and SSBs with >25 g sugar per portion, grouped by category.

Figure 3 displays the percentages of each SSB category rated as high sugar or high calorie in accordance with the three different evaluation methods. On the basis of the Chilean warning label criteria, only 6.2% of SSBs were classified as high-calorie drinks; among them, 14.0% of protein drinks and 5.1% of coffee and tea were high-calorie drinks. By contrast, 69.8% of SSBs were classified as high-sugar drinks; among them, 82.2% of fruit drinks and 74.8% of protein drinks were classified as high sugar. In accordance with the WHO guideline, more than half of all soft drinks contained >25 g sugar per portion, and more than 40.0% of coffee and tea and fruit drinks had >25 g sugar per portion.

Table 5 presents the comparisons of energy and sugar content for 100 mL and one portion among different SSBs. Protein drinks contained higher kcal/100 mL than the other SSBs, and soft drinks contained fewer kcal/100 mL than the other SSBs. However, protein drinks, soft drinks, and coffee and tea contained similar energy per portion, and fruit drinks contained less energy than the other drinks. Fruit drinks contained more sugar/100 mL than the other drinks. However soft drinks contained more sugar per portion than protein drinks and fruit drinks.

**Figure 4:**
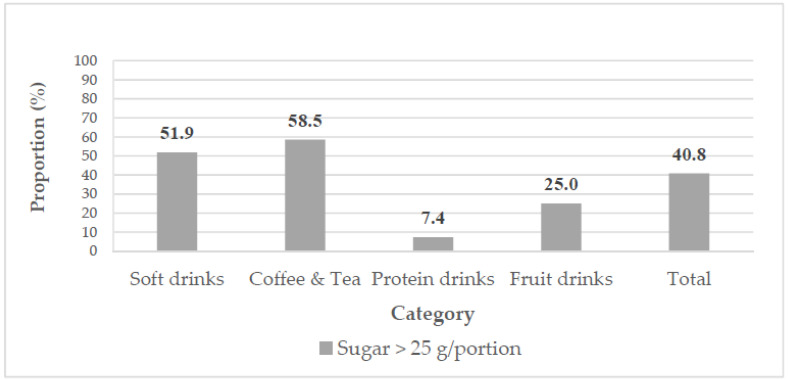
Distributions of >25 g sugar per portion in SSBs that were “not” classified as high sugar according to the Chilean criteria (*n* = 103).

As Figure 4 illustrates, we examined the sugar content of 103 drinks that were “not“ classified as high sugar according to the Chilean criteria. Among these drinks, 40.8% contained >25 g sugar per portion, including more than half of the soft drinks and coffee and tea drinks. Only 7.4% of protein drinks contained >25 g sugar per portion.

## 4. Discussion

In this study, nearly 20% of SSBs contained more than one serving. Soft drinks and coffee and tea that contained only one serving had higher energy per serving and sugar per serving than those containing >1 serving. Among protein drinks and fruit drinks, no significant differences in energy per serving or sugar per serving between portions containing one serving and >1 serving were found. However, the total energy and sugar per portion among different types of SSBs with >1 serving were significantly higher than SSBs with only one serving. Hence, people may make choices on the basis of the per-serving energy content and sugar content on the nutrition fact label and misjudge the serving size without realizing it. Most people may drink the whole bottle (portion) of a beverage, not only one serving. High SSB intake is related to the portion size consumed. The Australian Health Survey on adult nutrition found that the top 10% of SSB consumers drink more than 1 L/day [34]. Chepulis et al. noted that SSBs sold in larger containers (500 or 600 mL) can be consumed all at once, even if the nutrition label recommends two or three servings per portion, thereby leading to increased sugar intake [19]. Previous studies have verified that limiting the package size of beverages is an effective method to reduce sugar intake [35,36]. An Australian study revealed that a 375 mL limit on SSBs tended to reduce energy intake, weight gain, and economic burden [37].

In this study, the median energy per serving for all SSBs was 116 kcal, which was lower than that in China (140 kcal per serving) [38]. The median sugar per serving ratio for all SBBs was 21.65 g per serving, which was similar to results from surveys conducted in New Zealand, Australia, Canada, and the UK [19] but lower than results from China (42.1 g per serving) [38]. A great variation in serving sizes was observed among SSBs. We found 47 different serving sizes ranging from 50 to 600 mL. In Taiwan, the reference for serving sizes is considered to be established by people’s dietary patterns and common food package capacities [32]. Nevertheless, serving sizes often differed among the various SSB categories and were even inconsistent within SSB categories. This finding was consistent with those of surveys conducted in New Zealand, Australia, Canada, and the UK [19]; among 1070 food items in an Australian supermarket [39]; and 361 snacks and 246 drinks in a Taiwanese study [40]. Although consumers are interested in standardizing and controlling their intake, these variations are confusing and make it difficult to understand nutrition labels and choose proper foods.

Roberto and Khandpur noted that most nutritional education interventions designed to promote healthier eating habits use serving size and nutrition labels on packaged foods to educate consumers about the energy and nutrient content; however, it is difficult for consumers to use nutrition labels to calculate total energy when a package of food contains more than one serving [41]. Other studies have also reported that the use of serving size and per-serving nutritional information is poorly understood by consumers [42,43]. In the United States and Canada, serving sizes are regulated to reflect the portion sizes consumed [44,45]. In the United States, for certain products that are larger than a single serving but that could be consumed in one sitting or multiple sittings, manufacturers must provide dual-column labels to indicate the amount of energy and nutrients on both a per-serving and per-package or per-unit basis [45]. In Canada, single-serving prepackaged foods containing up to 200% of the reference amount for that food must label the entire container as one serving [44]. A UK study indicated that the portion sizes that consumers consumed were significantly higher than the serving sizes in some food groups, especially for unhealthy foods, such as potato chips and popcorn, meaning that consumers were consuming more energy than a single serving and too much sugar, fat, saturated fat, and sodium [46]. Hence, the serving size regulation might not be helpful for the general population who do not consume a standard serving size. In order to clarify the energy and nutrient contents of SSBs for consumers, the United States and Australia have mandated or recommended that the serving size should equal the entire package size [25,45]. In Taiwan, it was announced that from October 2020, regulations on labels of freshly made beverages will be amended to indicate the total volume, energy, and sugar content [47]. With this strategy, the total energy and sugar per portion relative to the energy and sugar per serving can be easily understood and used.

Previous studies have reported that FOP labels on food packaging, such as the Chilean warning labels, can provide consumers with easy-to-understand nutritional information [18,48]. In this study, the Chilean warning criteria and the WHO sugar guideline were used to evaluate different types of SSBs. A total of 62.5% of soft drinks were classified as high-sugar drinks under the Chilean criteria, which was a lower proportion than the other types of SSBs (coffee and tea, protein drinks, and fruit drinks), as shown in Figure 3. However, when using the WHO guideline (25 g sugar per portion), 56.9% of soft drinks contained excessive sugar, which was higher than the other types of SSBs. This is because the portion size of soft drinks is significantly larger than that of other types of SSBs. Although the median energy ratio (32.4 kcal/100 mL) was lower for soft drinks compared to other SSBs, the portion sizes for soft drinks are typically larger, so the energy content per portion ends up being similar to other SSBs (coffee and tea and protein drinks). A similar trend was observed for sugar content; the median sugar content in soft drinks was 7.7 g/100 mL, which was significantly lower than that in protein drinks and fruit drinks, but sugar content per portion was significantly higher than that in protein drinks and fruit drinks. Approximately half of soft drinks that were not considered as high sugar according to the Chilean criteria contained >25 g sugar per portion. This was because the portion size of soft drinks was larger than that of protein drinks and fruit drinks. Soft drinks, such as carbonated drinks, are popular worldwide [49] and are the major source of added sugars in some countries [22]. Carbonated sodas are often sold in large portion sizes (e.g., 1–2 L) and are less expensive than other types of SSBs. These larger portion sizes distort consumers’ perceptions of what a normal serving should be and how much they should consume [19].

Tea and coffee are both excellent sources of antioxidants and are therefore perceived as healthier choices than other SSBs [50]. A meta-analysis found that drinking two to three cups of tea per day reduced the risk of mortality, cardiac death, coronary artery disease, stroke, and type 2 diabetes mellitus with increment, and beneficial associations were also found for several cancers, skeletal, cognitive, and maternal outcomes [51]. Green tea supplement has significant protective effects against chronic diseases [52]. Coffee has been associated with a likely reduced risk of breast, colorectal, colon, endometrial and prostate cancer, cardiovascular disease, and type 2 diabetes [53]. However, the high sugar content of commercial sugar-sweetened coffee and tea cannot be ignored. In particular, sugar-sweetened tea is the principal SSB consumed by Taiwanese adolescents, and the mean frequency of sugar-sweetened tea consumption increased from 4.3 times/week in 1993–1996 to 5 times/week in 2010–2011 [54]. In this study, the sugar content of coffee and tea was 7.4 g/100 mL, which was significantly lower than that of other types of Taiwanese SSBs and similar to that of coffee and tea sold in the US (6.2–7.0 g/100 mL) [55] and Beijing (7.0–7.5 g/100 mL) [38]. Nevertheless, because of the large portion size of coffee and tea, the total sugar content per portion was significantly higher than that of protein drinks and fruit drinks. Additionally, because of the large portion size, approximately 60% of coffee and tea drinks that were considered acceptable under the Chilean criteria exceeded the WHO sugar guideline. Such high sugar content can have negative health effects. A previous study found that high sugar-sweetened coffee or tea intake was associated with poor dietary quality and high serum uric acid values among Taiwanese youth [56].

Our study found that 74.8% of protein drinks were classified as high sugar under the Chilean criteria, which was a higher proportion than soft drinks and coffee and tea. However, protein drinks, coffee and tea, and fruit drinks all contained similar sugar per portion, which was lower than that of soft drinks. Only 27.1% of all protein drinks and 7.4% of protein drinks considered acceptable under the Chilean criteria contained >25 g sugar per portion. This might be because protein drinks had higher sugar concentrations and smaller portion than the other SSBs. Protein drinks, such as dairy products, provide a variety of nutrients, including protein, calcium, riboflavin, and vitamin B12 [57,58]. They are also considered beneficial to bone health, cardiovascular health, and muscle and nerve function [57,59]. According to the findings of the 2013–2016 and 2017–2020 Nutrition and Health Survey in Taiwan, the average calcium intake is significantly lower than the recommendation; 80%–90% of people consume less than one serving of dairy products each day, and people aged 7–65 years old consume an average of only 0.3–0.5 servings per day [3,60]. Accordingly, consuming two servings of dairy drinks was emphasized by the Dietary Guideline of Taiwan [61]. Several national dietary guidelines worldwide also recommend that adults consume two to four servings of dairy products daily [62]. A systematic review found that flavored milk was used to promote milk consumption and meet recommended dietary allowances (RDA) for vitamin D and calcium, despite the risk of increased sugar and energy consumption [63]. Accordingly, strategies should be devised to reduce sugar in dairy foods without affecting consumer acceptance [64]. Evaluating sweetened dairy products on a per-portion, as opposed to sugar/100 mL, basis will help those who need to consume more dairy drinks make better choices. Warning labels can encourage manufacturers to develop more reduced-sugar products [65].

In our study, 82.2% of fruit drinks were classified as high sugar under the Chilean criteria, and fruit drinks had the highest sugar concentration among SSBs. A total of 42.2% of fruit drinks contained >25 g sugar per portion, thus exceeding the WHO guideline (Figure 3). Furthermore, 25.0% of fruit drinks that were not classified as high sugar under the Chilean criteria were considered high sugar by WHO standards. Fruit drinks, especially 100% fruit juices, are commonly perceived as healthier option than other SSBs. Collin et al. noted that although sugars in fruit juices are naturally occurring as opposed to added sugars, the effects of natural and added sugars on physiological metabolism may be similar [66]. Excessive juice consumption increases energy intake and increases the risk of dental caries and weight gain [67]. A previous study found that higher consumption of fruit drinks had the same effect on increasing mortality among older US adults as other SSBs [66]. Nevertheless, a systematic review revealed that fruit juice consumption in adults did not significantly affect weight [68]. Another meta-analysis indicated that 100% fruit juice consumption was not associated with higher cardiovascular risk [69]. Fruit juice or drinks may not be as deleterious as other SSBs for those who wish to control their body weight, but they should still be consumed in moderation [70].

A study reported that although food reformulation may be an area of debate, it still has an important effect on diet quality [71]. Another study that analyzed the nutritional composition of food and beverage product supply in Chile before implementation of the Chilean Food Labeling and Advertising Law in June 2016 found that only a few products (<2%) would have avoided at least one warning label as a result of product formulation [65]. By contrast, another study revealed substantial 58% and 94% reductions in “high sugar” warning labels for beverages and milk/milk-based drinks, respectively, after implementation of the Chilean law [13]. Another study focusing on the Australia/New Zealand Health Star Rating System initiative noted 1.5% decrease in energy in products assigned a Health Star Rating label; however, the Health Star Rating label was voluntarily implemented by less than 5% of local food suppliers, so these results might be biased [72,73]. A UK study revealed that children’s yogurt products exceeded the UK Food Standards Agency’s medium boundary for sugar, and a high proportion of children’s cereal bars exceeded the high boundary for sugar [74]. Other types of voluntary actions can improve the nutritional quality of foods [75,76,77]. Overall, the influence of voluntary initiatives on food supply has not been well characterized. Therefore, the FOP label strategy could be a good strategy to make the industry engage in food reformulation. Further studies should clarify how food reformulation affects the dietary quality of the population through implementation of an FOP label strategy for SSBs.

This study has some limitations. First, the sample might not include all commercial SSBs of less than 600 mL in Taiwan. However, we did our best to collect data from the major supermarkets and convenience stores in Taiwan. Second, we used total sugar, not added sugar, to evaluate Taiwan SSBs because only total sugar is available on Taiwanese nutrition labels. Further research on promoting FOP nutrient warning label policies should be conducted. Finally, our data were derived from the nutrient amounts reported by food manufacturers on packaging nutrition labels and were not obtained through laboratory assessments.

## 5. Conclusions

We observed a large variation in serving sizes on nutrition labels of SSBs. Total energy and sugar content per portion in SSBs exceeded that of a single serving. This might mislead people to choose SSBs with large portions but small serving sizes, resulting in overconsumption of calories and sugar. Mandatory actions, such as the implementation of high-sugar warning labels, might induce manufacturers to reformulate SSBs. For SSBs with volumes suitable for individual consumption, because of the great variation in portion size, nutrition labeling and warning criteria should be determined on a per-portion, and not a per-serving, basis. Evaluating SSBs on sugar/portion rather than sugar/100 mL will help consumers make better choices. The majority of SSBs in Taipei, Taiwan, could be classified as “high sugar” or “high calorie” according to international criteria, which would have detrimental impact on the health and weight management of Taiwanese consumers. Hence, the government should examine whether changes to labeling, including FOP warnings, would force manufacturers to reduce the amount of sugar added to beverages or encourage consumers to buy fewer unhealthy SSBs.

## Figures and Tables

**Table 1:** Coded components of food packaging information.

Coding Content	Coding Standard
General information	Product name, brand name
Beverage category	Soft drink, coffee and tea, protein drink, fruit drink
Portion size	Total volume of drink
Nutrition fact label	Serving size; number of servings; calories; and protein, total fat, saturated fat, carbohydrate, sugar, and sodium contents

**Table 2:** Nutritional information for different SSBs ^1,2^.

	Energy	Sugar	Carbohydrates	Protein	Fat	Saturated	Sodium
	(kcal)	(g)	(g)	(g)	(g)	Fat (g)	(mg)
Total	116 (84.2–160.0)	21.0 (15.5–27.0)	22.8 (17.5–30.0)	1.2 (0.0–4.6)	0.6 (0.0–3.9)	0.2 (0.0–2.1)	5.20 (28.0–97.0)
Soft drinks	92.2 (68.9–121.5) ^c^	21.7 (16.0–29.8)	22.7 (17.3–30.3)	0.0 (0.0–0.0) ^c^	0 ^c^ (0.0–0.0) ^c^	0 (0.0–0.0) ^b^	33.0 (18.5–76.3) ^b^
Coffee and Tea	115.0 (84.6–172.1) ^b^	21.0 (16.7–26.1)	23.0 (18.1–29.0)	1.3 (0.0–3.9) ^b^	2.0 (0.0–4.7) ^b^	1.2 (0.0–3.8) ^b^	69.0 (34.5–102.5) ^b^
Protein drinks	141.0 (110.0–187.0) ^a^	20.5 (12.4–25.7)	22.0 (16.7–30.0)	5.2 (3.6–6.4) ^a^	3.8 (2.2–5.6) ^a^	1.4 (0.6–2.6) ^a^	68.0 (30.0–106.0) ^a^
Fruit drinks	96.0 (67.9–145.7) ^bc^	21.8 (14.6–34.5)	24.0 (16.9–36.8)	0.0 (0.0–0.5) ^c^	0.0 (0.0–0.0) ^c^	0.0 (0.0) ^b^	38.0 (26.0–64.0) ^b^
*p* value	<0.001 **	0.09	0.69	<0.001 **	<0.01 *	<0.001 **	<0.001 *

^1^ Data are presented as the median (IQR). * *p* < 0.05 ** *p* < 0.001, obtained using the Kruskal–Wallis test. ^2^ Each column a > b > c.

**Table 3:** Variations in serving size and portion size among different SSBs ^1,2^.

	N (%)	Serving Size (mL)		Portion Size (mL)		*p* Value ^3^
		Median (Range)	CV %	Median (Range)	CV %	
Soft drinks	72 (21.1)	300.0 (165.0–600.0) ^a^	19.0	337.5 (192.0–600.0) ^a^	33.6	<0.001
Coffee and Tea	117 (34.3)	275.0 (100.0–600.0) ^b^	25.1	300.0 (150.0–600.0) ^b^	35.9	<0.001
Protein drinks	107 (31.4)	250.0 (50.0–450.0) ^c^	36.7	250.0 (100.0–517.0) ^c^	38.3	0.125
Fruit drinks *p* value ^4^	45 (13.2)	250.0 (125.0–500.0) ^c^ <0.001	36.5	250.0 (125.0–580.0) ^c^ < 0.001	42.7	0.250

^1^ CV: coefficients of variation; Range: Minimum–Maximum; ^2^ Each column a > b > c; ^3^ *p* obtained using the Wilcoxon signed rank test; ^4^ *p* obtained using the Kruskal–Wallis test.

**Table 4:** Energy and sugar content in different numbers of servings among different types SSBs ^1^.

		Energy (kcal)		Sugar (g)	
	*n*	Per Serving	Per Portion	Per Serving	Per Portion
**Soft drinks**					
1 serving	40	102.0 (78.0–131.8)	102.0 (78.0–131.8)	24.7 (18.9–31.8)	24.7 (18.9–31.8)
>1 serving	32	80.20 (50.0–107.0)	160.4 (100.0–214.0)	19.8 (12.0–26.0)	39.6 (24.1–52.0)
*p* value		0.003 *	<0.001 **	0.006 *	<0.001 **
**Coffee and Tea**					
1 serving	89	138.0 (95.8–192.0)	138.0 (95.8–192.0)	21.8 (17.4–27.0)	21.8 (17.4–27.0)
>1 serving	28	82.4 (58.2–113.3)	180.0 (148.4–227.8)	19.8 (12.7–22.4)	39.7 (30.4–45.2)
*p* value		<0.001 **	0.002 *	0.020 *	<0.001 **
**Protein drinks**					
1 serving	103	141.0 (109.0–188.8)	141.0 (109.0–188.8)	20.4 (12.4–25.7)	20.4 (12.4–25.7)
>1 serving	4	139.0 (124.5–153.5)	304.0 (268.0–1000.0)	21.7 (14.2–27.3)	43.4 (28.5–228.6)
*p* value		0.850	0.001 *	0.599	0.006 *
**Fruit drinks**					
1 serving	42	95.0 (68.4–148.8)	95.0 (68.4–148.8)	21.5 (15.2–34.9)	21.5 (15.2–34.9)
>1 serving	3	116.0 (90.6–116.5)	232.0 (181.0–233.0)	25.0 (19.3–26.3)	50.0 (38.5–52.5)
*p* value		1.000	0.026 *	0.964	0.031 *

^1^ Data are presented as the median (IQR). * *p* < 0.05 and ** *p* < 0.001, obtained using the Mann–Whitney *U* test.

**Table 5:** Description of energy and sugar content among different SSBs ^1,2^.

Categories	Energy (kcal)		Sugar (g)	
	Per 100 mL	Per Portion	Per 100 mL	Per Portion
Soft drinks	32.4 (26.1–41.1) ^c^	119.5 (89.7–161.7) ^a^	7.7 (6.1–10.0) ^bc^	29.0 (21.2–39.9) ^a^
Coffee and Tea	40.0 (32.8–53.1) ^b^	148.4 (99.0–200.0) ^a^	7.4 (6.5–8.1) ^c^	24.5 (18.2–30.0) ^ab^
Protein drinks	59.5 (52.0–65.2) ^a^	141.6 (110.0–193.0) ^a^	8.3 (5.2–10.2) ^b^	20.8 (13.2–26.4) ^b^
Fruit drinks	43.0 (37.4–46.4) ^b^	96.0 (71.5–153.6) ^b^	9.8 (8.4–10.6) ^a^	22.0 (16.1–35.6) ^b^
*p* value	<0.001 **	<0.001 **	<0.001 **	<0.001 **

^1^ Data are presented as the median (IQR). ** *p* < 0.001, obtained using the Kruskal–Wallis test. ^2^ Each column a > b > c.
